# Duration of mechanical ventilation on the result of diaphragmatic function in weaning

**DOI:** 10.1186/cc9580

**Published:** 2011-03-11

**Authors:** HB Qiu, W Guo, Y Yang

**Affiliations:** 1 Zhongda Hospital, Southeast University, Nanjing, China

## Introduction

To investigate the influence of duration of mechanical ventilation on the diaphragmatic function.

## Methods

Patients included in this study were those mechanically ventilated for at least 24 hours and were preparing to wean from December 2008 to December 2009 in the ICU of Nanjing Zhong-Da Hospital. Patients, according to the duration of mechanical ventilation, were divided into group A (ventilated less than 3 days) and group B (ventilated more than 3 days). A 30-minute spontaneous breathing test (SBT) was carried out on the patients satisfying the weaning permission. Indices of diaphragm function such as electrical activity of diaphragm (Edi), neuromuscular strength index (NMS), neuromechanical coupling (NMC) and neuroventilatory coupling (NVC) at 0, 5 and 30 minutes of SBT were monitored.

## Results

Forty-four patients were included finally, of whom 19 patients (43.2%) were ventilated more than 3 days (group B), while the average duration of mechanical ventilation was 6.2 ± 3.9 days. Twenty-five patients were ventilated less than 3 days (group A), whom had an average duration of mechanical ventilation for 2.2 ± 0.7 days. There was no significant difference in Edi, NMS, NMC or NVC at 0 minutes of SBT between the two groups. Edi and NMS in group B were 20 ± 11 μV and 571 ± 338 μV·cpm at 5 minutes of SBT, which were both largely more than group A (16 ± 8 μV and 387 ± 208 μV·cpm, *P *< 0.05). Then, NMC and NVC had no significant difference. At SBT 30 minutes, Edi and NMS in group B both were significantly higher than group A (23 ± 11 μV vs. 15 ± 8 μV, 598 ± 309 μV·cpm vs. 362 ± 224 μV·cpm, *P *< 0.05). Whereas NVC in group B (20 ± 12 ml/μV) was lower than group A (35 ± 21 ml/μV, *P *< 0.05).

## Conclusions

The contractility and endurance of diaphragm decreased in patients whom were ventilated more than 3 days at 30 minutes of SBT. It seemed that an incremental duration of mechanical ventilation could exacerbate diaphragm dysfunction, which might be one of the important factors leading to failed weaning.

